# The combination of tumor mutational burden and T‐cell receptor repertoire predicts the response to immunotherapy in patients with advanced non–small cell lung cancer

**DOI:** 10.1002/mco2.604

**Published:** 2024-06-05

**Authors:** Yalun Li, Liyan Ji, Yingqian Zhang, Jiexin Zhang, Alexandre Reuben, Hao Zeng, Qin Huang, Qi Wei, Sihan Tan, Xuefeng Xia, Weimin Li, Jianjun Zhang, Panwen Tian

**Affiliations:** ^1^ Department of Pulmonary and Critical Care Medicine State Key Laboratory of Respiratory Health and Multimorbidity West China Hospital of Sichuan University, Precision Medicine Key Laboratory of Sichuan Province Chengdu Sichuan China; ^2^ Lung Cancer Center/Lung Cancer Institute West China Hospital, Sichuan University Chengdu Sichuan China; ^3^ Geneplus‐Beijing Institute Beijing China; ^4^ Departments of Bioinformatics and Computational Biology University of Texas MD Anderson Cancer Center Houston Texas USA; ^5^ Department of Thoracic/Head and Neck Medical Oncology University of Texas MD Anderson Cancer Center Houston Texas USA; ^6^ Department of Pulmonary and Critical Care Medicine West China Hospital, West China School of Medicine, Sichuan University Chengdu Sichuan China; ^7^ Department of Genomic Medicine University of Texas MD Anderson Cancer Center Houston Texas USA; ^8^ Lung Cancer Genomics Program University of Texas MD Anderson Cancer Center Houston Texas USA; ^9^ Lung Cancer Interception Program University of Texas MD Anderson Cancer Center Houston Texas USA

**Keywords:** advanced non–small cell lung cancer, clonality, immunotherapy, T‐cell receptors, tumor mutational burden

## Abstract

Tumor mutational burden (TMB) and T‐cell receptor (TCR) might predict the response to immunotherapy in patients with non–small cell lung cancer (NSCLC). However, the predictive value of the combination of TMB and TCR was not clear. Targeted DNA and TCR sequencing were performed on tumor biopsy specimens. We combined TMB and TCR diversity into a TMB‐and‐TCR (TMR) score using logistic regression. In total, 38 patients with advanced NSCLC were divided into a discovery set (*n* = 17) and validation set (*n* = 21). A higher TMR score was associated with better response and longer progression‐free survival to immunotherapy in both the discovery set and validation set. The performance of TMR score was confirmed in the two external validation cohorts of 225 NSCLC patients and 306 NSCLC patients. Tumors with higher TMR scores were more likely to combine with *LRP1B* gene mutation (*p* = 0.027) and top 1% CDR3 sequences (*p* = 0.001). Furthermore, *LRP1B* allele frequency was negatively correlated with the top 1% CDR3 sequences (*r* = –0.55, *p* = 0.033) and positively correlated with tumor shrinkage (*r* = 0.68, *p* = 0.007). The TMR score could serve as a potential predictive biomarker for the response to immunotherapy in advanced NSCLC.

## Graphical abstract

Targeted DNA and TCR sequencing were performed on tumor biopsy specimens from ICI‐treated NSCLC patients. We combined TMB and TCR diversity into a TMB‐and‐TCR (TMR) score. The predictive value and prognostic role of TMR was assessed in NSCLC patients receiving immunotherapy. The performance of TMR score was confirmed in the two external validation cohorts from MD Aderson Cancer Center (MDACC).

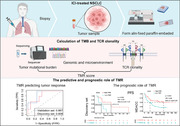

## 1 INTRODUCTION

Immunotherapy is increasingly being used in the treatment of NSCLC, and the clinical need to predict the response to immunotherapy is emerging. Immunotherapy biomarkers can be classified into tumor neoantigen‐related factors (e.g., tumor mutational burden [TMB], *STK11* gene mutation) and microenvironment factors (e.g., programmed cell death‐ligand 1 [PD‐L1], gene expression profile [GEP] score).^1^ Although PD‐L1 expression is a requirement in the guideline of the clinic usage of mono‐immunotherapy, it is clear that PD‐L1 expression is not a perfect biomarker. Several biomarkers based on the gene expression of immune cells were extensively studied such as GEP, IFNG, antigen processing and presenting machinery (APM), immune infiltration score, tumor immunogenicity score (TIGS), tumor immune dysfunction and exclusion (TIDE), and their prognostic performance were ranging from 0.38 to 0.75, not reaching the clinic requirement.^2^, ^3^ Therefore, it is still a challenge for the identification of immunotherapy biomarkers.

As an intratumor feature, TMB can predict response to immunotherapy and patients’ survival in cancers such as non–small cell lung cancer (NSCLC) and bladder cancer.^4^, ^5^ Meanwhile, studies have shown that high TMB (≥10 mut/Mb) failed to act as a universal predictive and prognostic biomarker for immunotherapy across all cancers at either 10 mut/Mb or an optimized cutoff value.^4^, ^5^ The ability of tumors generating neoantigens influences the effects of checkpoint inhibitors, while simultaneously, infiltrating T cells around the tumor determine the immune response to checkpoint inhibitors. Thus, combining intratumor and extratumor factors may enhance the accuracy of predictive and prognostic biomarkers.

Recently, biomarker combinations have mainly focused on TMB and PD‐L1 or GEP. For NSCLC, TMB and microenvironment factors (typically PD‐L1) were independent predictive factors of response to immunotherapy.^6^ Specifically, the combination of TMB^high^ and GEP^high^ exhibited better performance for predicting response to mono‐immunotherapy than TMB or GEP alone in NSCLC.^7^ However, the correlation of TMB and GEP exhibited controversial role across pan‐tumors,^8^ limiting their usage in clinics. For other combined biomarkers, TMB and PD‐L1, the ratio of benefited patients was 1.8‐fold higher in TMB^high^ PD‐L1^high^ patients than in TMB^high^ PD‐L1^low^ patients.^6^ Although TMB was independent of PD‐L1 expression in most cancers, these two factors were positively correlated in neuroendocrine cancer and endometrial cancers.^9^ Importantly, most tumors were without available tissues for both TMB and PD‐L1 testing.^10^ Thus, independent and ubiquitous biomarkers of predicting the response to immunotherapy are urgently needed.

Tumor genomic mutation is an original driven for progression and evasion, either by self‐driven or interaction with surrounding immune cells. High TMB of tumor generates neoantigen, and induces a high immune response. Tumor microenvironment, specifically, immune cells play a synergistic role in tumor evasion. T‐cell‐mediated cytotoxic antitumor activity depends on the interaction between T‐cell receptors (TCRs) and major histocompatibility complexes, along with the co‐inhibitory programmed cell death protein‐1 (PD‐1) and PD‐L1 molecules.^11^ Zhang et al. reported that TCR clonality correlated with pathologic response to anti‐PD‐1 therapy in NSCLC.^12^ Highly expanded clones in treatment‐naive blood could predict the pathologic response in patients with NSCLC receiving chemoimmunotherapy.^13^ However, the predictive role of the combination of TMB and TCR status has not yet been reported in NSCLC.

Herein, we assessed the predictive role of the combination of TMB and TCR clonality in NSCLC patients receiving immunotherapy. TCR characterization was explored in patients with high TMB and TCR clonality. Using integrative analysis, we revealed that T‐cell clonality, which interacted with genomic gene mutations, was correlated with tumor size, and could predicted the outcome. Our study provided evidence that incorporating tumor and immune assessment might improve the predictive performance of immunotherapy outcomes in NSCLC.

## 2 RESULTS

### 2.1 Patient characteristics

Of the 38 patients, a median age at diagnosis was 61 years (range, 55–67 years), 26 (68%) were males and 12 (32%) were females, 19 (50%) were smokers and 19 (50%) were nonsmokers. Twenty‐five patients (66%) were diagnosed with lung adenocarcinoma, 12 patients (31%) were squamous cell carcinoma, and 1 patient (3%) was diagnosed with unclassified NSCLC. Nine patients (24%) were diagnosed with stage III and 29 (76%) were stage IV. Eleven patients (29%) were PD‐L1 negative, 22 patients (58%) were PD‐L1 positive, and 5 patients (13%) were unknown PD‐L1 status. Thirty‐one patients (82%) received combination therapy with immune checkpoint inhibitors (ICIs) and chemoradiotherapy or antiangiogenic therapy, and 7 patients (18%) received mono‐immunotherapy. The demographic and clinical variables such as age, sex, and immunotherapy were matched between the discovery set (*n* = 17) and validation set (*n* = 21). The demographic and clinical characteristics of patient sets are summarized in Table 1.

**TABLE 1:** Demographic characteristics of patient sets.

	Discovery Set (*N* = 17)	Validation Set (*N* = 21)	Total (*N *= 38)	*p‐value*
Diagnosed age (years)				
Age (median [IQR])	57.00 [54.00, 63.00]	64.00 [55.75, 69.50]	61.00 [55.00, 67.00]	0.174
Gender (%)				
Male	13 (76)	13 (62)	26 (68)	0.337
Female	4 (24)	8 (38)	12 (32)	
Smoke status (%)				
Smoker	7 (41)	12 (57)	19 (50)	0.328
Nonsmoker	10 (59)	9 (43)	19 (50)	
Histology (%)				
Adenocarcinoma	10 (59)	15 (71)	25 (66)	0.591
Squamous	6 (35)	6 (29)	12 (31)	
unclassified NSCLC	1 (6)	0 (0)	1 (3)	
TNM stage (%)				
III	6 (35)	3 (14)	9 (24)	0.249
IV	11 (65)	18 (86)	29 (76)	
Kind of immunotherapy (%)				
Anti‐PD‐1	17 (100)	20 (95)	37 (97)	1.000
Anti‐PD‐L1	0 (0)	1 (5)	1 (3)	
Response (%)				
Partial Response	2 (12)	9 (43)	11 (29)	0.120
Stable	10 (58)	8 (38)	18 (47)	
Progression	5 (30)	4 (19)	9 (24)	
Number of prior lines (%)				
1	14 (82)	18 (86)	32 (84)	1.000
≥2	3 (18)	3 (14)	6 (16)	
PD‐L1 status (%)				
Negative (< 1%)	6 (35)	5 (24)	11 (29)	0.109
Positive (≥1%)	11 (65)	11 (52)	22 (58)	
Unknown	0 (0)	5 (24)	5 (13)	

Abbreviations: IQR, interquartile range; NSCLC, non–small cell lung cancer; PD‐L1, programmed cell death‐ligand 1.

**FIGURE 1:**
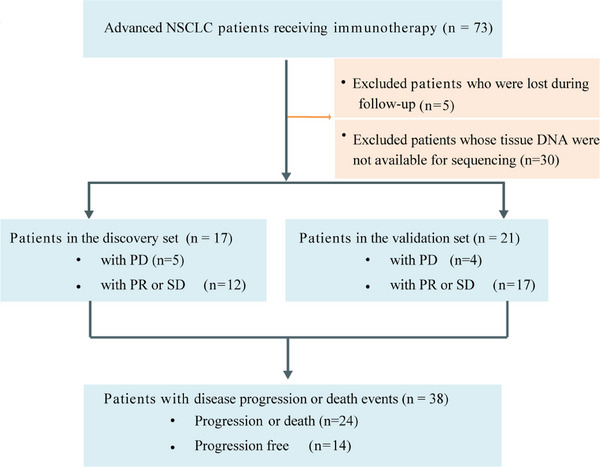
Study design and patients. Flowchart describing patient participation in this study. The diagram shows patients’ inclusion and exclusion, discovery and validation set, and the endpoint of this study.

The overall disease control rate was 76.3% (29 of 38 patients), whereas nine patients had disease progression (Figure S1A). Median tumor reduction was −13.3% (range, −74.6% to 183.3%) (Figure S1B). The median follow‐up duration was 223 days (Figure S1C). In the discovery set (*n* = 17), 12 patients obtained a partial response (PR) or stable disease (SD). In the validation set (*n* = 21), 17 showed a PR or SD (Figure 1).

### 2.2 Higher TMR scores were associated with better response to immunotherapy

**FIGURE 2:**
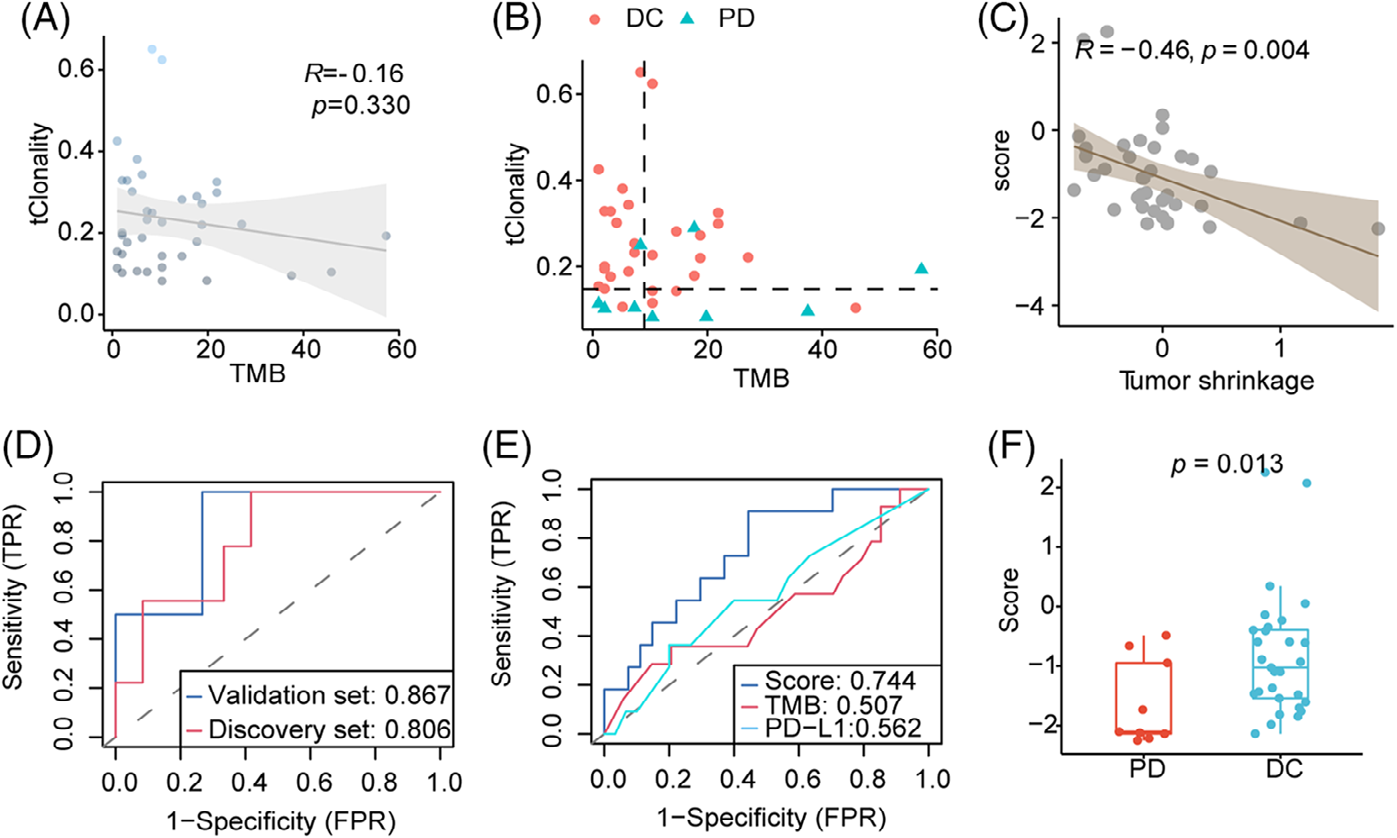
TMR score was associated with better response to immunotherapy in patients with advanced non–small cell lung cancer. (A) Pearson correlation analysis of tumor mutational burden (TMB) and T‐cell receptor (TCR) clonality in the tumor samples. (B) TMB and TCR clonality distribution is classified by disease control (DC) and disease progression (PD). Patients with DC are represented in red, while those with PD are shown in cyan. (C) A higher TMR score (regression score of the combination of TMB and TCR clonality) was correlated with tumor shrinkage based on Pearson correlation analysis. (D) The predictive performance of the TMR score for distinguishing the response of patients receiving immunotherapy in the discovery group (*n* = 17) and the validation group (*n* = 21). (E) Receiver operating characteristics curve for the association of TMR score, TMB, and programmed cell death‐ligand 1 (PD‐L1) with objective response (DC/PD) in all patients (*n* = 38). (F) Bar plot depicting the TMR score in tumors of patients with DC or PD. TMB, tumor mutational burden. TCR, T‐cell receptor. TMR, TMB, and TCR score.

Through sequencing matched tumor tissue‐blood DNA, we identified 452 somatic mutations with an average depth of 1203x in 38 patients. The median TMB was 8 mut/Mb (range, 1–55 mut/Mb). TMB was not significantly correlated with clonality (*r* = −0.16, *p* = 0.330; Figure 2A), suggesting an independent relationship between TMB and clonality. Of the 29 patients with disease control, 8 patients had high TMB (≥10 mut/Mb) and high clonality (≥the median of TMB) (Figure 2B). To further explore clinic relevance of TMB or clonality, we analyzed the correlation of these markers with clinicopathological characterization and commonly used clinic markers. Clonality was not correlated with patient age, TMB, or PD‐L1status (Figure S2A), while it was significantly correlated with tumor shrinkage (*r* = −0.45, *p* = 0.005; Figure S2B). Continuous TMB and PD‐L1 values were not associated with tumor reduction (Figure S2B).

TMB and TCR diversity was combined into a TMB‐ and‐TCR (TMR) score using logistic regression. Of the 38 patients, the TMR score was negatively correlated with tumor shrinkage (*r* = −0.46, *p* = 0.004; Figure 2C). For the TMR score, the area under the receiver operating characteristics curve (AUC) for predicting tumor response was 0.806 in the discovery group and 0.867 in the validation group (Figure 2D), respectively. Compared with TMB or PD‐L1 alone, the TMR score was marginally better for predicting disease control (AUC = 0.744 for TMR score, AUC = 0.507 for TMB, AUC = 0.562 for PD‐L1; *p* = 0.100; Figure 2E). For comparing the diagnostic performance of TMR with the combination of TMB plus PD‐L1 or clonality plus PD‐L1, the results showed that the TMR was slightly superior to these combinations (AUC = 0.664 for TMB plus PD‐L1; AUC = 0.708 for clonality plus PD‐L1, respectively; Figure S2C). Additionally, the TMR score was higher in patients with disease control than in those with disease progression (*p* = 0.013; Figure 2F).

### 2.3 TMR score predicted PFS in patients receiving immunotherapy

**FIGURE 3:**
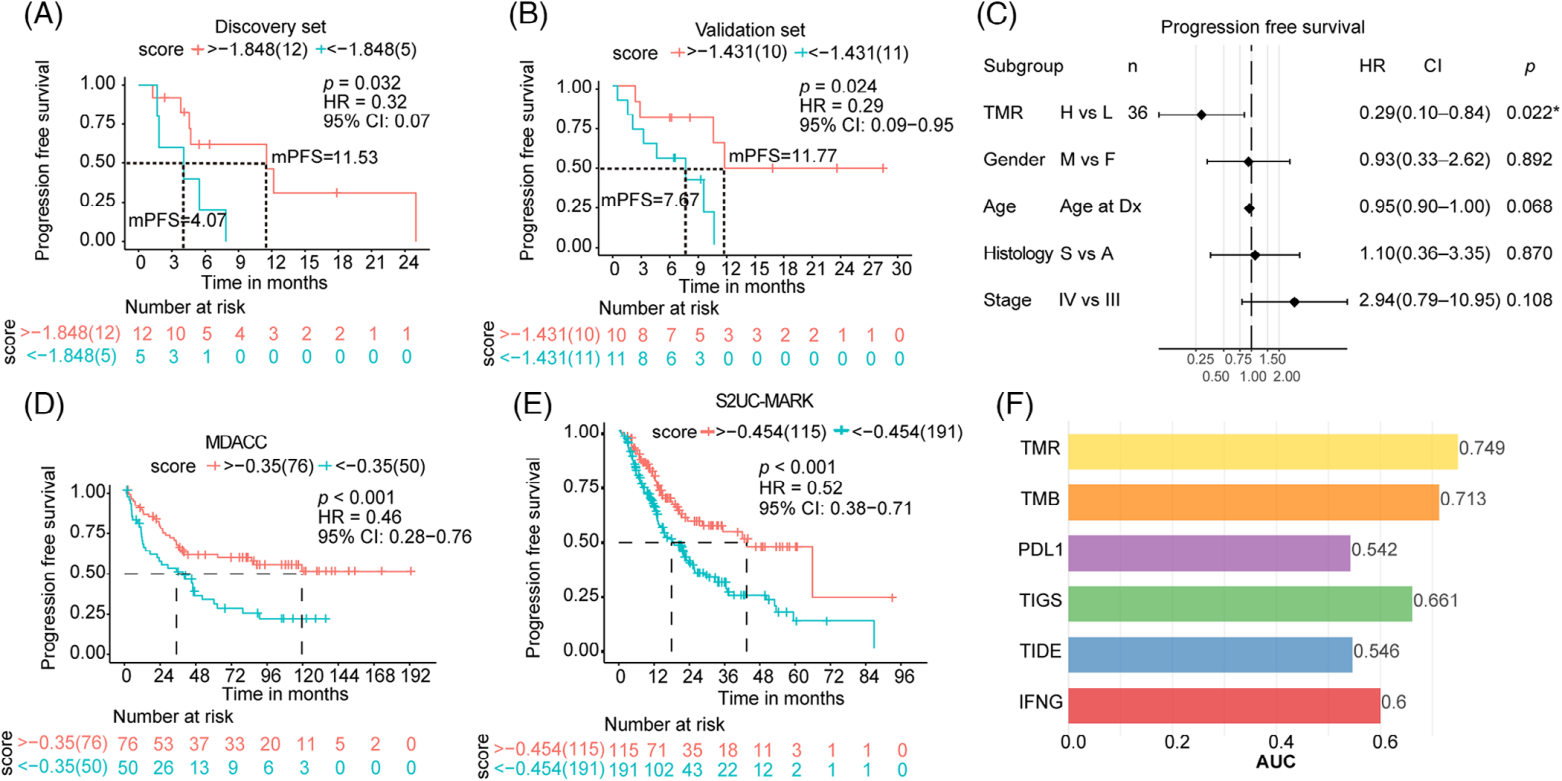
TMR score was associated with better PFS of patients with advanced non–small cell lung cancer receiving immunotherapy. Kaplan–Meier plots showing progression‐free survival (PFS) in the high‐ versus low‐TMR score groups in the (A) discovery group and (B) validation group. TMR scores represent the combination of tumor mutational burden and T‐cell receptor clonality. (C) Multivariate Cox model for the association between TMR score and PFS, after adjusting for clinical features (gender, age, histology, tumor stage). S: Squamous, A: Adenocarcinoma. (D) Kaplan–Meier plots showing progression‐free survival (PFS) in the high‐ versus low‐TMR score groups in the external validation cohort from MD Aderson Cancer Center (MDACC). (E) Kaplan–Meier plots showing overall survival (OS) in the high‐ versus low‐TMR score groups in the external validation set from the Stand Up To Cancer‐Mark Foundation (S2UC‐MARK) cohort. (F) Receiver operating characteristics curve for the association of TMR score, TMB, and programmed cell death‐ligand 1 (PD‐L1), TIGS, TIDE, and IFNG with overall survival (OS) in the external validation cohort (*n* = 65).

Progression‐free survival (PFS) was longer in patients with a high TMR score than in those with a low TMR score in the discovery group (median PFS was 11.53 months and 4.07 months for high TMR and low TMR score, respectively, hazard ratio [HR] = 0.32, *p* = 0.032; Figure 3A), as well as in the validation set (median PFS was 11.77 months and 7.67 months for high TMR and low TMR score, respectively, HR = 0.29, *p* = 0.024; Figure 3B). In a multivariate model, TMR score was found to be an independent predictive biomarker of PFS, when adjusting for age, histology, tumor stage, and sex (*p* = 0.022; Figure 3C).

### 2.4 Prediction model performance in external validation cohort

Using two external validations, we confirmed that patients with high TMR score exhibited longer survival than low TMR score group in MDACC cohort (hazard ratio [HR] = 0.46, *p* < 0.001; Figure 3D), as well as in the S2UC‐MARK cohort (HR = 0.52, *p* < 0.001; Figure 3E). Moreover, the TMR score demonstrated superior predictive efficacy compared with TMB, PD‐L1, integrative biomarkers such as TIGS, or other gene expression biomarkers including TIDE and IFNG alone in the S2UC‐MARK cohort (Figure 3F). These results facilitated the superior performance of the combination of TMB and T‐cell clonality in NSCLC.

### 2.5 TCR clonal expansion in the high‐TMR score group

**FIGURE 4:**
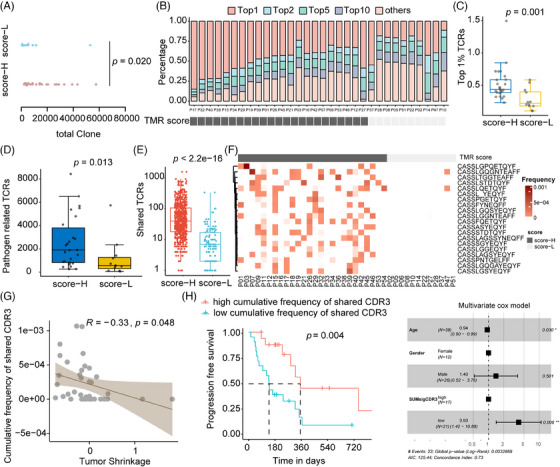
A Higher TMR score was associated with increased frequency of the most frequent and shared T‐cell receptor CDR3s. (A) Scatter plot demonstrating the total clone count in tumors with high versus low TMR scores. (B) Bar plot depicting the percentage of the most common clones (top 1%, top 2%, top 5%, top 10%) and others for each patient. Patients with high TMR scores are shown in dark gray, while those with low TMR scores are shown in light gray. (C) Comparison of the most frequent (top 1%) clone frequencies in the high‐TMR score versus low‐TMR score groups. A two‐sided Mann–Whitney *U* test was used to compare the groups. (D) Scatter plot of the number of pathogen‐related T‐cell receptors in patients with high TMR scores versus low TMR scores. (E) Scatter plot showing CDR3 shared between individuals within the high‐TMR score group or low‐TMR score group. (F) Heatmap of commonly shared CDR3s in patients with high TMR scores versus those with low TMR scores. Dark gray represents patients with a high TMR score, while light gray represents those with a low TMR score. (G) Pearson correlation analysis of the association between cumulative frequency of shared CDR3s and tumor shrinkage. (H) Kaplan–Meier and forest plots showing the association between progression‐free survival (PFS) and cumulative frequency of shared CDR3s (high versus low frequency) in patients with advanced non–small cell lung cancer receiving immunotherapy.

To explore the underlying immune status of TMR groups, the TCR parameters were analyzed. We determined the total clone count, representing the number of infiltrating T cells around the tumor. The total clone count was higher in patients with high TMR scores than in those with low TMR scores (*p* = 0.020; Figure 4A). Of the total TCR complementarity‐determining region (CDR)3 clones, the most frequent (top 1%) TCR clones were found in 43.17% (23.65% to 84.89%) of patients with a high TMR score and in 24.19% (11.22% to 58.54%) of patients with a low TMR score (Figure 4B). The most frequent CDR3 clonotypes were determined for each patient (Figure S3). The frequency of the most common (top 1%) TCR clones was significantly higher in patients with a high TMR score (*p* = 0.001; Figure 4C) than in those with a low TMR score. We also observed a greater abundance of pathogen‐associated TCRs detected by GLIPH2 in the high‐score group than in the low‐score group (*p* = 0.013; Figure 4D).

As shown in Figure 4E, patients with high TMR scores shared a higher number of CDR3s than those with a low TMR score (*p* < 0.001). The shared CDR3 clonotypes among 20 patients were found to be significantly enriched in high‐TMR score group patients (Figure 4F). The frequency of shared CDR3s was 30% (8/26) to 50% (13/26) in the TMR high‐score group patients, which was significantly higher than in the low‐score group patients (1/12; two‐sided Fisher's test, *p *= 0.027). The frequency of these shared CDR3s was negatively correlated with tumor shrinkage (*r* = −0.33, *p* = 0.048; Figure 4G). Furthermore, higher clone frequency of these shared CDR3s was associated with longer PFS in univariate and multivariate models (*p *= 0.004 and 0.008, respectively, *n* = 38; Figure 4H). These results suggested the activated immune existed in patients with high TMR score and might be a key determinator for response to immunotherapy.

Then the inducer of these highly frequency or shared CDR3 clonotype was explored. As shown in Figure S4A, B, both the most frequent TCRs and the shared TCRs have the common CDR beta motif CASS*EQYF. Through searching the McPAS‐TCR database, we noted that the most frequent (top 1%) TCRs accounted for 1.42%, 0.17%, and 0.31% in pathogen‐related, cancer‐related, and autoimmune/allergy‐related CDR3s, respectively (Figure S4C). Shared TCRs were found in the high‐TMR score group, especially 20 recurrent CDR3s. Of these, two were cancer‐related CDR3 betas (as deposited in the database),^14^, ^15^ while four were pathogen‐associated CDR3s,^16^, ^17^, ^18^, ^19^ and six were autoimmune‐linked CDR3s^20^, ^21^, ^22^, ^23^, ^24^ (Figure S4D). The underlying factor for these immunotherapy‐common shared TCRs might be partially induced by endogenous antigens.

### 2.6 Genomic mutation allele frequency was associated with TCR clonal expansion

**FIGURE 5:**
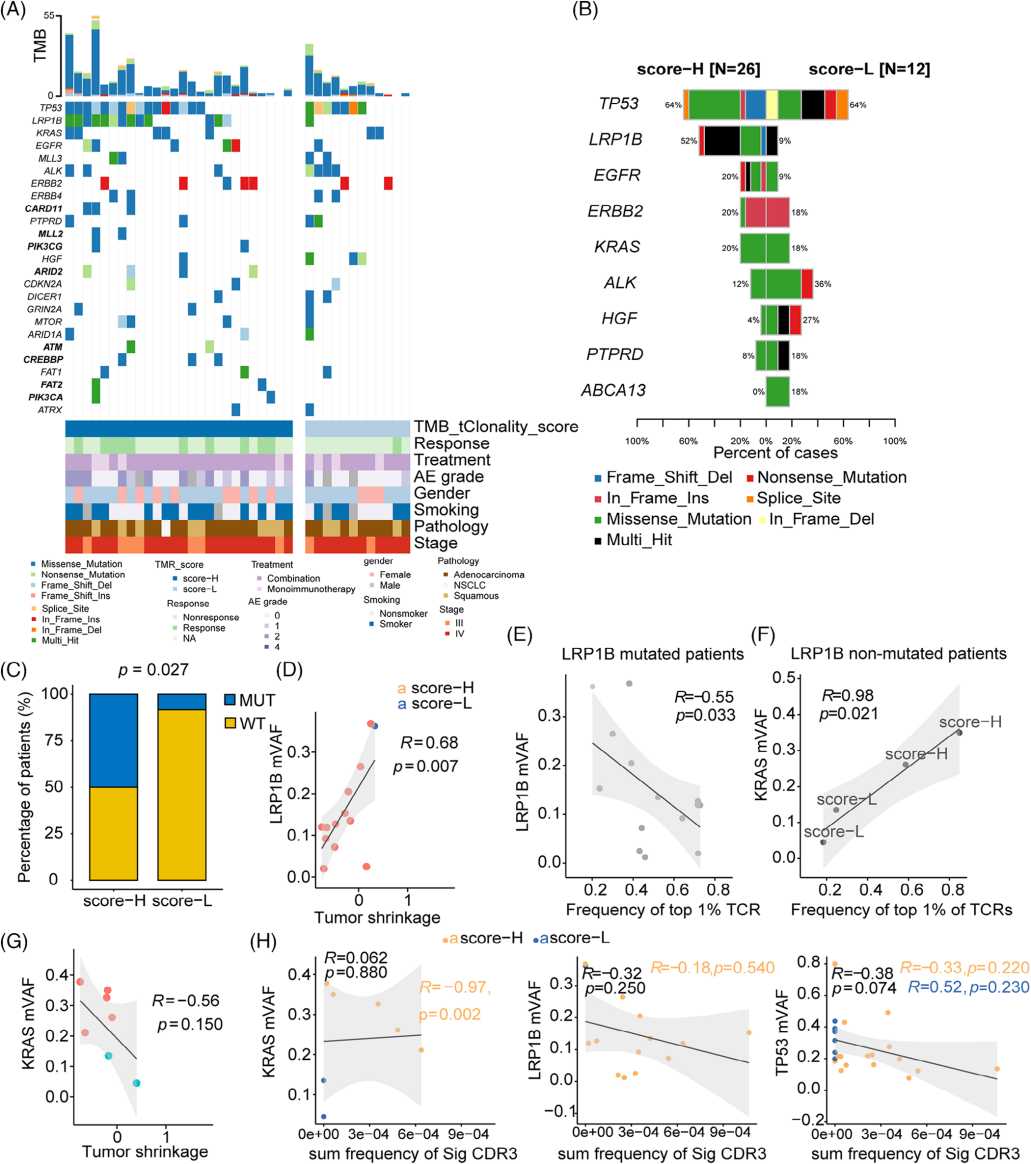
Genomic features linked to high and low TMR scores in patients with advanced non–small cell lung cancer receiving immunotherapy. (A) Oncoprint depicting the highly recurrent genes in our study. The individual column represents each patient, organized by patients with an objective response below. Nonsynonymous tumor mutational burden is shown in a bar plot at the top. Clinical features, including objective response, treatment, grade of adverse effect(s), sex, smoking, pathology, and stage are presented at the bottom. (B) Bar plot showing gene frequencies in patients with high TMR score (TMR‐H) or low TMR score (TMR‐L). (C) Bar plot depicting the percentage of patients with mutant and wild‐type *LRP1B*. A two‐sided Fisher's test was used to compare frequencies in the high‐ and low‐TMR score groups. (D) Pearson correlation analysis of the association between maximal variant allele frequency (mVAF) of *LRP1B* and tumor shrinkage. Patients with high TMR scores are shown in orange, while those with low TMR scores are depicted in blue. (E) Correlation between the most frequent (top 1%) CDRs and *LRP1B* mVAF in patients with *LRP1B* mutations. (F) Pearson correlation analysis of the association between the most frequent (top 1%) CDRs and *KRAS* mVAF in patients with wild‐type *LRP1B*. (G) Correlation of *KRAS* mVAF with tumor shrinkage. (H) Pearson correlation analysis of the association between the cumulative frequency of shared CDR3s and *KRAS* mVAF, *LRP1B* mVAF, and *TP53* mVAF. Each point represents the Pearson correlation coefficient for one patient, with black representing the coefficient of all patients with specific mutations. Orange represents the coefficient of patients in the high‐TMR score group with specific gene mutations, whereas blue represents the coefficient of patients in the low‐TMR score group with specific gene mutations.

To illustrate the genomic characterization associated with TMR, the driver genes were compared between high‐TMR and low‐TMR groups. The recurrent genes were distinct in high‐TMR and low‐TMR patients (Figure 5A). *CARD11*, *MLL2*, *PIK3CG*, *ARID2*, *ATM*, *CREBPP*, *FAT2*, and *PIK3CA* were exclusive mutated genes in the high‐TMR score group with no significances. The mutation frequencies for the high‐TMR score and low‐TMR score groups are shown in Figure 5B. *TP53* missense and frameshift mutations occurred frequently in patients with a high‐TMR score, whereas multiple mutations, splice site mutations, and nonsense mutations were preferentially enriched in patients with a low TMR score (Figure 5B). *LRP1B* was the only mutated gene that occurred significantly and more frequently in the high‐TMR score group than in the low‐TMR score group (two‐sided Fisher's test, *p* = 0.027; Figure 5B, C). Nonsense and frameshift deletions of *LRP1B* were known to be loss‐of‐function mutations.^25^ 90.5% of (19/21) missense mutations of *LRP1B* were predicted to be pathogenic or likely pathogenic mutations by FATHMM‐MKL^26^ (Table S1).

In the external S2UC‐MARK cohort, *LRP1B* mutation preferably occurred in the TMR‐H group (Figure S5A). The TMB was higher in mutant *LRP1B* than wild‐type *LRP1B* either in TMR‐H subgroup (*p* = 0.042; Figure S5B), or across ICI‐treated patients (*p* = 6.8 × 10^−7^; Figure S5C). To illustrate the relationship of TMB and *LRP1B*, the DNA mismatch repair genes *MSH6*, *MLH1*, *MSH2*, *POLE* that led to the high TMB in solid tumors were selected.^27^ *MSH6* and *MLH1* were co‐mutant with *LRP1B* in S2UC‐MARK cohort (both *p* < 0.05; Figure S5D). Consistently, a positive correlation of *LRP1B* gene expression with *MSH6* or *MLH1* mRNA levels was observed (Figure S5E), suggesting the positive relationship of *LRP1B* and TMB, one of the TMR components. Differentially expressed genes (DEGs) were identified between *LRP1B* mutant and *LRP1B* wild‐type patients (Figure S5F). Thirty DEGs were enriched in CXCR chemokine receptor binding, chemokine activity, and chemokine receptor binding pathways (Figure S5G). *LRP1B* mutant patients showed the activation of neutrophils immune cell infiltration, especially hN1 and hN5 subtypes estimated by *fgsea* using RNAseq (Figure S5H). These data indicated the *LRP1B* linked to TMR by co‐occurrence with MSH6/MLH1 and the activation of chemokine‐induced activation of neutrophils.

### 2.7 The interaction of genomic and microenvironment factors

Finally, the interaction of tumor genomic and microenvironment basis underlying the TMR was investigated. In patients with *LRP1B* mutations, the maximum variant allele frequency (mVAF) of *LRP1B* was significantly associated with tumor shrinkage (*r* = 0.68, *p* = 0.007; Figure 5D), and a negative correlation between the most frequent (top 1%) TCRs and mVAF (*r* = −0.55, *p* = 0.033; Figure 5E), indicating the *LRP1B* gene might affect the tumor response to immunotherapy‐based treatment. For patients with *LRP1B* ^−^ *KRAS* ^+^ mutations, mVAF of *KRAS* was positively correlated with the most frequent (top 1%) TCRs (*r* = 0.98, *p* = 0.021; Figure 5F), suggesting the positive role of *KRAS* mutation on the immune cell clonality. However, *KRAS* mVAF was not associated with tumor shrinkage (Figure 5G). These results of *KRAS* indicated the role of *KRAS‐*mutation‐induced immune activation in response to immunotherapy might be limited. Regarding shared CDR3s, neither *LRP1B* nor *KRAS* mutations were associated with shared CDR3s (Figure 5H). Additionally, the mVAF of *KRAS* was >0.2% in high‐TMR score group patients and was significantly correlated with shared CDR3s found in the group (*r* = −0.97, *p* = 0.002). For mutant *TP53*, mVAF was negatively correlated with the shared CDR3s, although the correlation did not reach statistical significance (*r *= −0.38, *p* = 0.074). The mVAF of *LRP1B* was marginally negatively correlated with influenza‐associated and yellow fever virus‐associated CDR3s (Figure S4E). It indicated the mutations of driver genes had a distinct role in response to immunotherapy, and *LRP1B* mutations, rather than *KRAS* or *TP53*, were more likely to participate in the pathogen‐induced immune activation that responses to immunotherapy.

## 3 DISCUSSION

Our study demonstrated that the TMR score might predict patients who would benefit from immunotherapy and higher TMR scores were correlated with better PFS. Additionally, we also revealed that the most frequent TCRs and shared TCRs both contribute to tumor shrinkage.

Previous studies have mostly focused on single biomarker, either tumor or microenvironment factors, for predicting the response to immunotherapy. The combination of tumor and microenvironment biomarkers has also been explored and reported to have better accuracy.^7^ Cristescu et al.^7^ evaluated the combination of TMB and GEP in the predicting clinical response to checkpoint blockade immunotherapy which focused on advanced solid tumors and melanoma across 22 tumor types. Using TMB combination of APM genes, termed as APM score, showed a superior predictive accuracy than single TMB or gene expression biomarkers.^2^ However, this needs the integration of sequencing at both DNA and RNA levels. In the current study, we showed the TMB was independent of TCR, and their incorporation could serve as a tumor predictive and prognostic biomarker. Contrary to the previous study,^28^ we observed no significant correlation between TMB and clonality in our study. This discrepancy might be ascribed to the inconsistent observations of different tumor stages. Specifically, Reuben's study included stage I‐III NSCLC, whereas 76% of patients were advanced NSCLC (stage IV) in our study. Thus, we combined TMB with TCR clonality in patients with NSCLC receiving immunotherapy and found that the combination was useful for predicting response to treatment and predicting outcome. Two external immunotherapy‐based datasets validated that a combination of tumor genomics and T‐cell environment could predict the response and outcome of immunotherapy, irrespective of sequencing methodology. In line with the previous reports,^2^ the combination of both genomic and immune‐infiltrate (TMR score) performed better response prediction ability than tumoral mutations or microenvironmental single gene expression biomarkers (TIGS, TMB, TIDE, PDL1, IFNG) under the immunotherapy including anti‐PD‐1 monotherapy and combination immunotherapy. Intriguingly, we revealed that the combination of the TMB and TCR burden from WES data as a score showed a similar prediction performance with WES plus RNA or DNA panel plus TCR, providing the potentially cost‐and‐effective application for the simultaneous detection of somatic changes and immune cell infiltrate.

In our study, we provided that the most frequent TCRs and shared TCRs both contribute to tumor shrinkage, suggesting that the expanded T‐cell clones might kill tumor cells. It has been reported that specific expanded T‐cell clones and CDR3 motifs are induced by oncogenic mutations, for example, *KRAS* or *ERBB2* ^29^, ^30^ and pathogen stimulations.^31^ However, we did not identify a typical motif in either the most frequent (top 1%) TCRs or shared TCRs, possibly because of the complex mutation spectrum (*EGFR*, or *KRAS*, or *ERBB2* co‐occurrent driver genes) in NSCLC.^32^, ^33^ Furthermore, immune‐related TCRs were more abundant than cancer‐related TCRs in both the most frequent and shared TCRs. These results suggested that immunotherapy partially induced external stimulation (pathogen)‐related or autoimmune‐specific T cells to kill tumor cells. Pathogen‐related TCRs were enriched in patients with a high TMR score. This observation was consistent with data suggesting that microbiota residing within and around tumors, including lung cancer, contribute to tumor growth and the response to immunotherapy.^34^ Novel TCRs related to immunotherapy in cancer were identified in our NSCLC patients. The role of most of these TCRs and their associated T‐cell subtypes are largely unknown. Thus, these results require further validation and exploration in the future.

Recent studies reported that TMB and mutations may work independently to impact antitumor activity of immune response.^28^ However, interactions among TMB, genomic DNA mutation, and T cells remain unknown. Higher TMB was associated with *LRP1B* mutation consistent with the previously reported study.^35^ This might be ascribed to the co‐occurrence of *LRP1B* with *MSH6*/*MLH1*, genes that related to hypermutation, according to observation from the external data. Except for its impact on the genomic alteration, *LRP1B* might be an immuno‐repressor. In our study, mutations in *LRP1B* were seen at increased frequency in patients in the high‐TMR score group. Additionally, higher mVAF of mutant *LRP1B* correlated with a lower clone frequency of the most frequent (top 1%) TCRs, suggesting that *LRP1B* loss‐of‐function has an activator role in the T‐cell immune response. These results were in line with the observation that *LRP1B* mutations might activate the antitumor immune cells and immune‐related pathways.^35^, ^36^ This may explain why patients with *LRP1B* mutations treated with immunotherapy had longer PFS as well as in the multicancer study by Brown et al.^25^ Concurrently, we observed a positive correlation between *KRAS* allele frequency and the most frequent TCR clone fraction in NSCLC. This result is in line with the previous report, increased fraction of shared TCR clones has been associated with increased *KRAS* mutation frequency in colorectal cancer.^37^ Together with observations for *TP53*, our findings suggested that tumor suppressor genes tended to suppress immune T cells, whereas oncogenic *KRAS* genes were more likely to induce T‐cell expansion. As 95% of solid tumors harbored more than one driver gene (typically 2.6 coding drivers),^38^ the immune status was possibly determined by multiple driver genes with mutual interactions. Thus, examining driver genes and the TCR repertoire might be an important next step in the comprehensive assessment of NSCLC and its microenvironment.

This study had several limitations, including a small sample size, use of multiple therapies (mono‐immunotherapy and combination chemoimmunotherapy), and no experimental evidence of gene mutation dose and TCR expansion. Nevertheless, our study was based on observations in real‐world data and provided results with potential clinical implications, suggesting that the TMR score might be a potentially useful clinical surrogate biomarker for TMB or PD‐L1 in some circumstances.

## 4 CONCLUSION

Our study demonstrated that the TMR score, which was a combination of TMB and TCR clonality, might have a potentially predictive role in patients with advanced NSCLC receiving immunotherapy. Additionally, we found the dose‐dependent interaction between the tumor and microenvironment T cells at the genomic and immune level in NSCLC tissue.

## 5 MATERIALS AND METHODS

### 5.1 Patient collection

Consecutive patients with advanced NSCLC (stage IIIA‐IVB) diagnosed at West China Hospital of Sichuan University from December 30, 2018, to September 24, 2021, were recruited. Patients received anti‐PD1/PD‐L1 therapy were included. Patients lost to follow‐up or unavailable for TCR sequencing or targeted DNA sequencing were excluded. This study was approved by the Ethics Committee of West China Hospital (no. 2021‐1451), and the project was performed in accordance with the Declaration of Helsinki as revised in 2013.

Seventy‐three patients (ages ranging from 44 to 88 years) with advanced NSCLC who received immunotherapy were retrospectively collected. Targeted DNA sequencing was available in 54 patients, whereas samples from 43 of these patients were successfully sequenced for T‐cell CDR3 beta, then five patients who lost follow‐up were excluded. Finally, 38 patients were included in our study. The discovery group consisted of 17 patients diagnosed with NSCLC before June 30, 2020. The validation set consisted of 21 patients diagnosed as NSCLC after June 30, 2020 (Figure 1).

### 5.2 Treatment evaluation

The radiological response of tumors was evaluated using computed tomography every 8−10 weeks, and the radiologist was independent and blinded. Disease progression was evaluated according to the immune‐related Response Evaluation Criteria in Solid Tumors Criteria.^39^ PFS was defined as the time from the date of treatment initiation until radiographic progression or death from any cause, whichever occurred first. Patients without radiographic disease progression on the date of the last follow‐up were classified as censored. Tumor shrinkage/reduction means the tumor size decreased from the time of diagnosis to the follow‐up CT scan.

### 5.3 Tissue tumor mutational burden assessment

Tissue TMB was assessed using targeted DNA sequencing. Briefly, genomic DNA was extracted from formalin‐fixed paraffin‐embedded (FFPE) samples of pretreatment biopsies using Maxwell FFPE Plus DNA Kit (Promega Biotech Co.). DNA (0.8−1 µg) was sheared into 200−250 bp fragments using a Covaris ultrasonicator (Covaris, LLC, Inc.). Libraries were constructed using the NEBNext Ultra DNA Library Prep Kit (NEB) with unique molecular identifier adaptors. Target enrichment of libraries was performed with a 1021‐gene panel, as previously reported.^40^ Sequencing was conducted using the Geneplus 2000 platform (Geneplus‐Suzhou Biomedical Engineering Corporation), with 100‐bp paired‐end reads. Low‐quality reads were filtered out from raw reads, and the reads were mapped into the hg19 human genome. Mutect2 and GATK were used to detect somatic single‐nucleotide variants and indels. Somatic variants were retained when the VAF was >1% and when ≥5 reads supported the variant. Patients with TMB (≥10 mut/Mb) were defined as TMB‐H,^4^, ^5^ while others were considered as TMB‐L group.

### 5.4 TCRβ sequencing

High‐throughput sequencing of CDR3 beta was performed on genomic DNA obtained from FFPE biopsy samples. The rearranged TCR beta was amplified by multiplex PCR primers, as previously reported.^41^ The DNA library was sequenced on the Geneplus 2000 platform (Geneplus‐Suzhou Biomedical. Engineering Corporation), with 100‐bp paired‐end reads. Down‐sampling to one million reads was performed for each sample to conduct further bioinformatical analysis. The number of unique CDR3 clones for each sample was defined as the total clone count. T‐cell clonality was calculated as follows (with *p*_i_ representing the percentage of rearrangement i and N being the total count of TCR rearrangements): Clonality = 1 − $\frac{\sum_{i\;=\;1}^{R}p_{i}lnp_{i}}{lnN}$. Pathogen‐associated TCRs were clustered using the grouping of lymphocyte interactions by paratope hotspots, version 2 (GLIPH2) algorithm,^18^ and McPAS‐TCR database.

### 5.5 Calculation of TMB and TCR clonality (TMR) score

Samples with both TMB and clonality values were used for further analysis. The TMR score was calculated for each NSCLC tissue sample using TMB continuous values combined with TCR clonality values and response to immunotherapy as categorical outcomes by logistic regression analysis. The function was calculated as glm (as.factor(Response) ∼ TMB + tClonality, data, family = binomial(“logit”) using the R *stats* package (v4.2.1). The optimal cutoff of the TMR score for PFS was determined by Cox hazard analysis using surv_cutpoint function with minprop = 0.3 parameter in the R survminer package.

Other combination factors (PD‐L1) were assessed by the method the same as TMR score. The prognostic performance shown as AUC was compared among TMR and TIDE, TIGS, IFN, and GEP of the Stand Up To Cancer‐Mark Foundation cohort (S2UC‐MARK).

### 5.6 External validation cohorts

The 225 early‐stage NSCLC patients from A. Reuben's study were used as external validation cohort.^28^ We evaluated the performance of TMR score using T‐productive clonality, PD‐L1 tumor H‐scores, and TMB in the external cohort. PFS was set as the primary endpoint.

The second external validation set was downloaded from S2UC‐MARK cohort (https://doi.org/10.5281/zenodo.7625517).^42^ Here, the TMB and the T_cell_burden based on WES data were used for the TMR calculation (*n* = 306) with the removal of three low‐quality samples. For the integration of mutation and expression analysis, we used 65 patients sequenced by WES and matched RNAseq. For gene expression markers, the TIDE score was assessed by TIDE software (http://tide.dfci.harvard.edu/).^43^ And the TIGS was calculated via Wang's method.^2^ Other gene expression biomarkers (INFG, GEP, and immune cell infiltrations) were provided by the supplementary materials of S2UC‐MARK study.^42^

### 5.7 Statistical analysis

AUC was calculated using the ROCit package. Variables were compared between high TMR‐score and low TMR‐score subgroups using the Mann–Whitney *U* test. Missing data were removed from the analysis. The correlations were determined by Pearson correlation coefficients. Two‐sided *p‐*values < 0.05 were considered significant. The multivariate Cox model was adjusted for age, histology, tumor stage, and gender. All analyses were performed and statistical images were created using R (version 4.1.0) software.

## AUTHOR CONTRIBUTIONS

Yalun Li conceived and designed the research, and wrote the manuscript. Liyan Ji performed all analyses and wrote the manuscript. Jiexin Zhang collected the data and performed all analyses. Alexandre Reuben, Hao Zeng, Qin Huang, Qi Wei, Jianjun Zhang, and Sihan Tan collected the data. Yingqian Zhang and Xuefeng Xia wrote the manuscript. Weimin Li and Panwen Tian obtained financial support and reviewed and edited the manuscript. Jianjun Zhang and Panwen Tian were the overall principal investigators who conceived the study and revision of the manuscript. All authors contributed to the article and approved the final version of the manuscript.

## CONFLICT OF INTEREST STATEMENT

Liyan Ji, Yingqian Zhang, and Xuefeng Xia are employees of Geneplus‐Beijing Institute company but have no potentially relevant financial or nonfinancial interests to disclose. The remaining authors declare no conflict of interest.

## ETHICS STATEMENT

The research was carried out according to the World Medical Association Declaration of Helsinki and was approved by the Ethics Committee of West China Hospital (approval number: 2021‐1451).

## Supporting information

Supporting Information

Supporting Information

## Data Availability

The data generated in this study are available from the corresponding author upon reasonable request.
