# Supplementary material for: The combination of tumor mutational burden and T‐cell receptor repertoire predicts the response to immunotherapy in patients with advanced non–small cell lung cancer
Source: MedComm (2020). 2024 Jun 5;5(6):e604. doi: 10.1002/mco2.604 (PMC11151154; doi:10.1002/mco2.604)
Supplement: Supplementary file 1 — Supporting Information [file MCO2-5-e604-s001.docx]

The combination of TMB and TCR repertoire predict the response and outcome of immunotherapy in advanced non-small cell lung cancer

Supplementary Materials

Supplementary figure legends


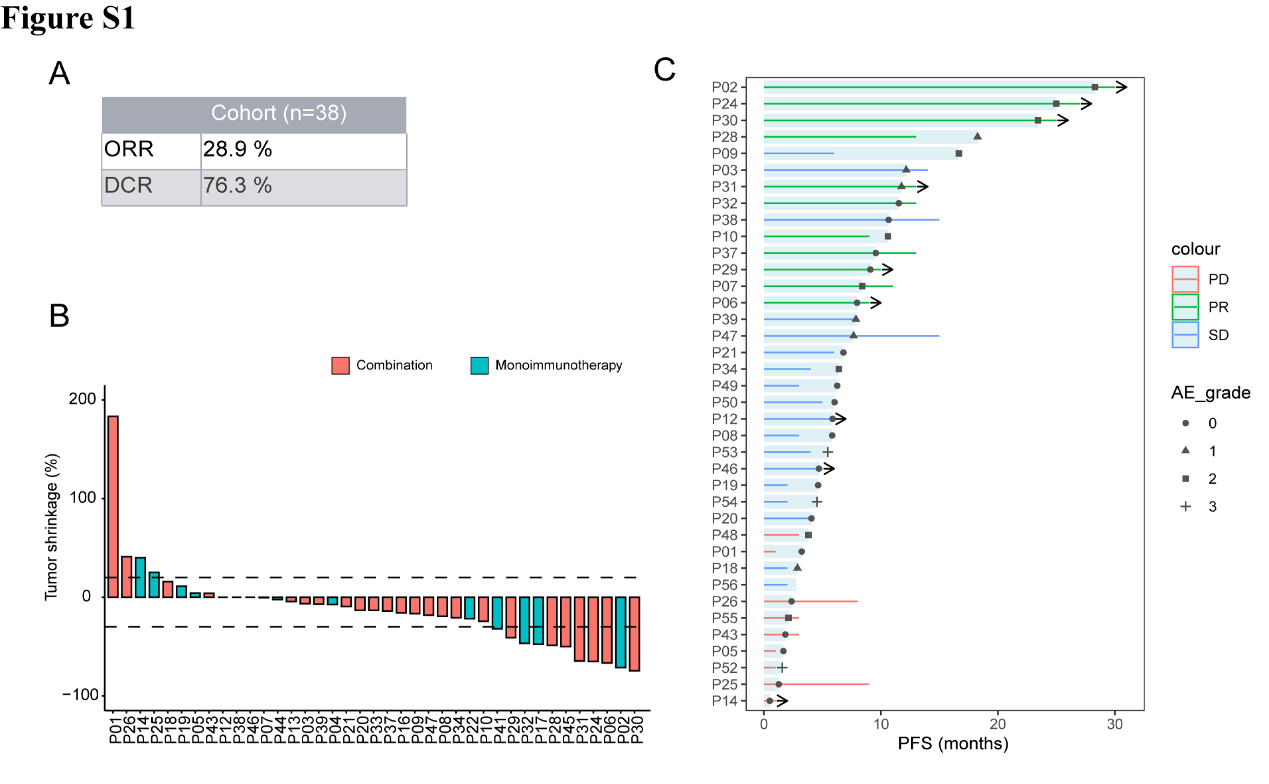


**Figure S1. Efficacy of immunotherapy in real world lung cancer patients. (A) Efficacy of immunotherapy in advanced NSCLC of our cohort.** (B) Swimmer plot of tumor shrinkage in our cohort. The patients receiving combination were colored red, while those given monoimmunotherapy were colored by green. (C) The spider plot showing PFS for each patient, and the response was colored for PD, PR, and SD as red, green, and blue, respectively. The adverse effects were also indicated by different symbols.


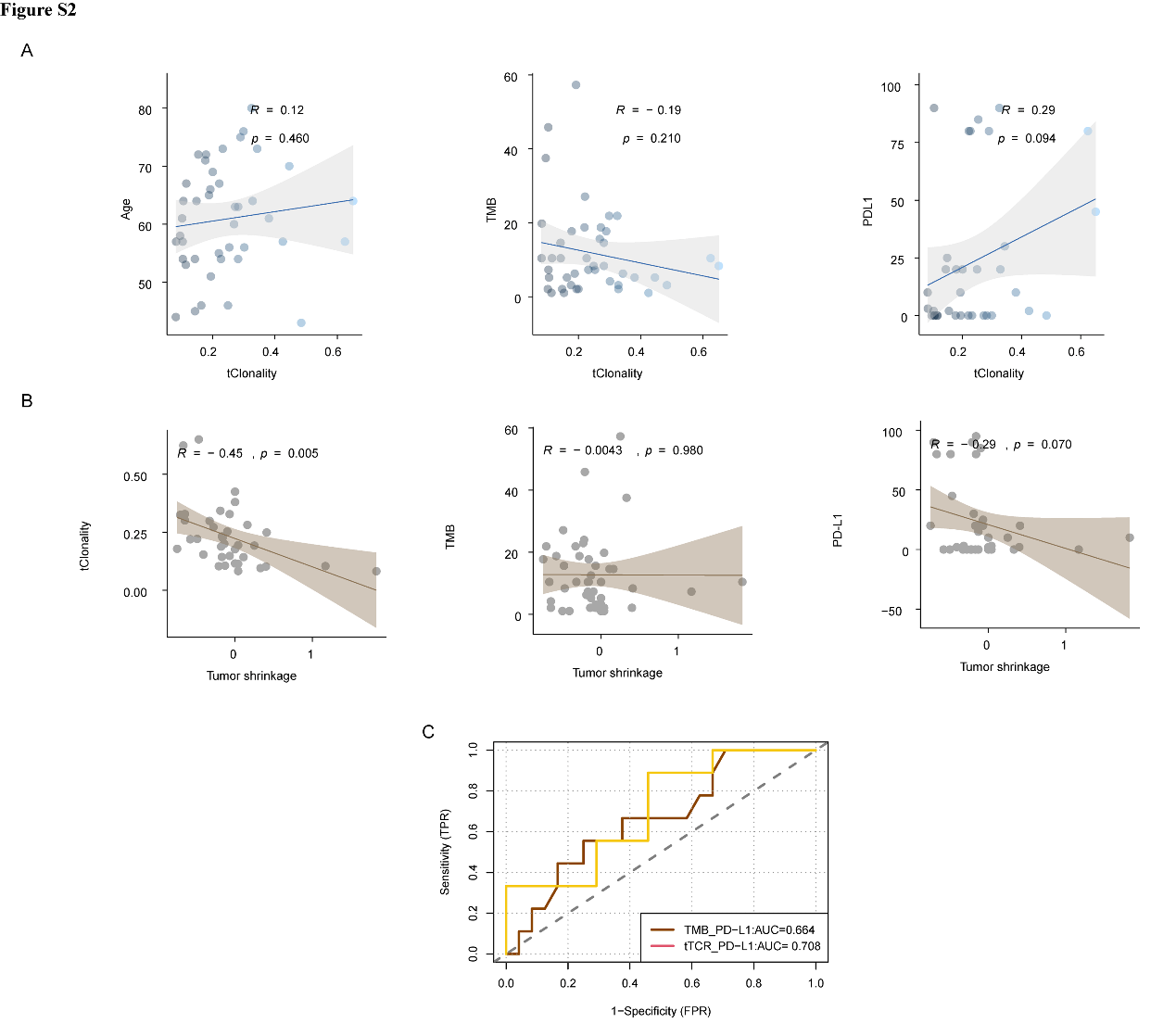


**Figure S2. The correlation of molecular and clinical features**. (A) Pearson correlation of tissue clonality with age, TMB, and PD-L1. (B) The correlation of tumor shrinkage with tissue clonality, TMB, and PD-L1. (C) Receiver operating characteristics curve for the association of clonality, TMB, and programmed cell death-ligand 1 (PD-L1) with objective response (DC/PD) in all patients (n = 38)


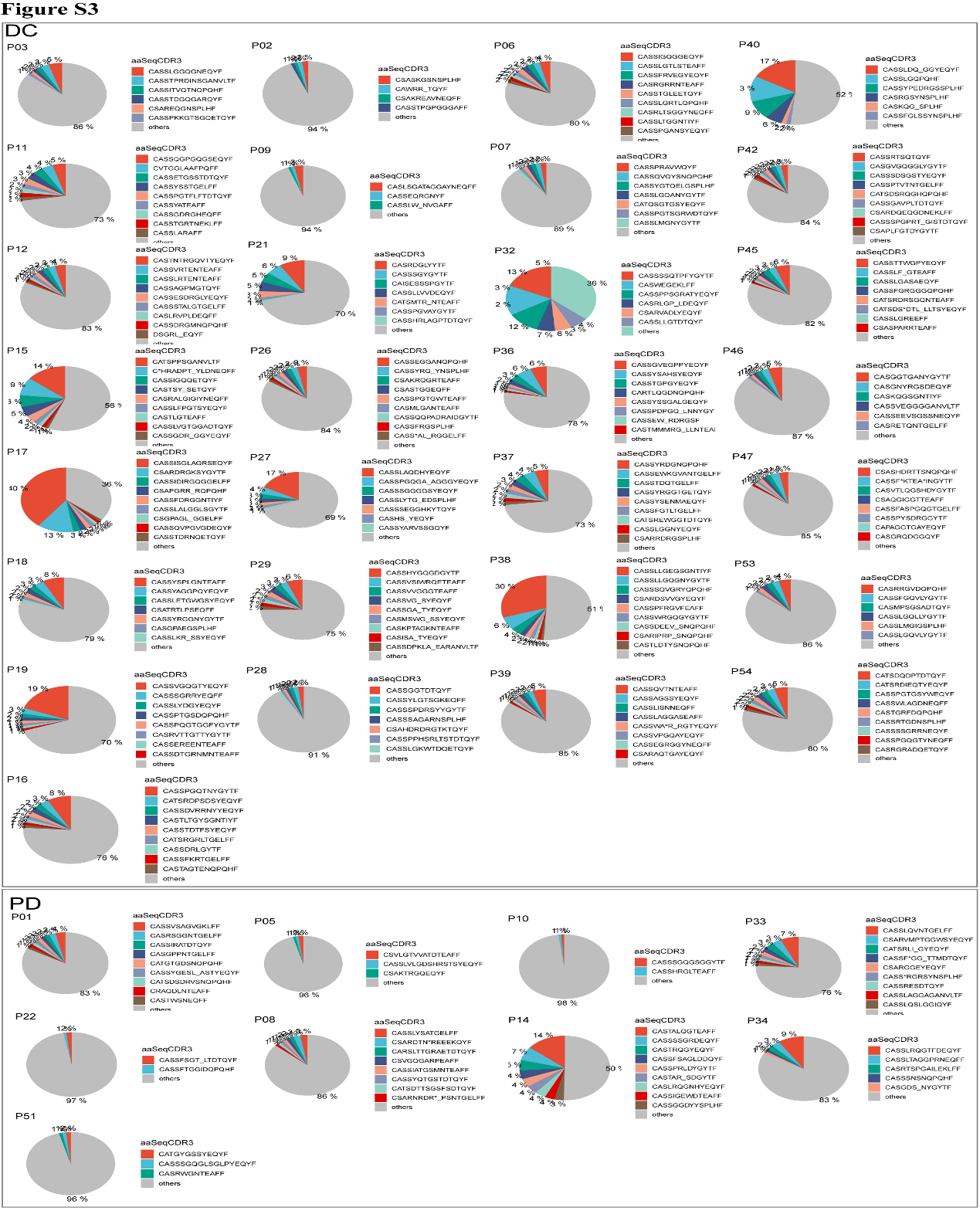


**Figure S3. The aaSeqCDR3 frequency in each patient treated with immunotherapy.**


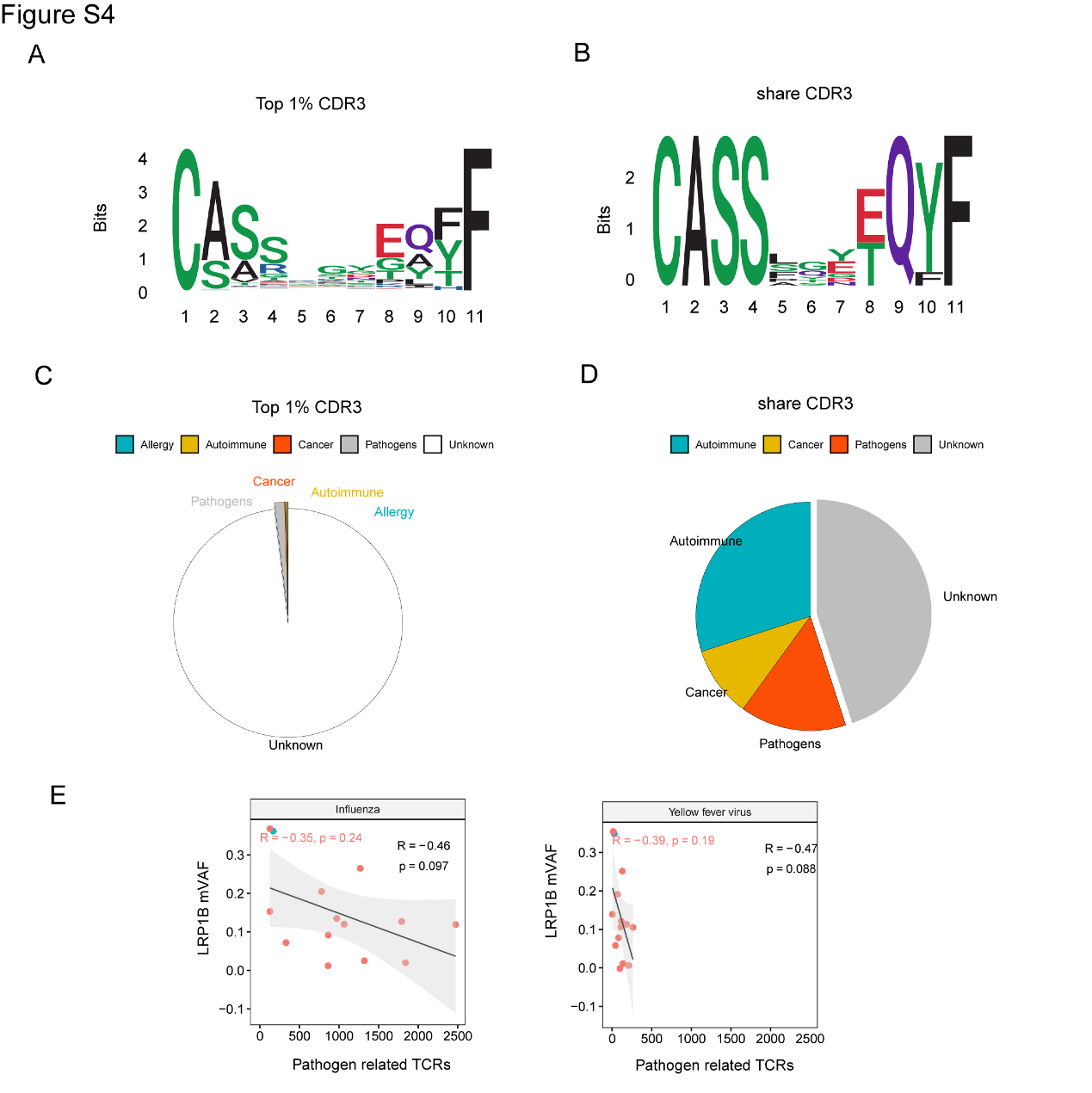


**Figure S4. The correlation of gene allele frequency and CDR3 clone frequency.** The motif sequence enriched in top 1% CDR3 in all patients (A) and 20 shared CDR3 in high-TMR score patients (B). The disease-associated CDR3 of top 1% CDR3 in all patients (C) and 20 shared CDR3 in high-TMR score patients (D). (E) The correlation of maximal variant allele frequency of LRP1B with virus-associated CDR3 fraction predicted by GLIPH2.


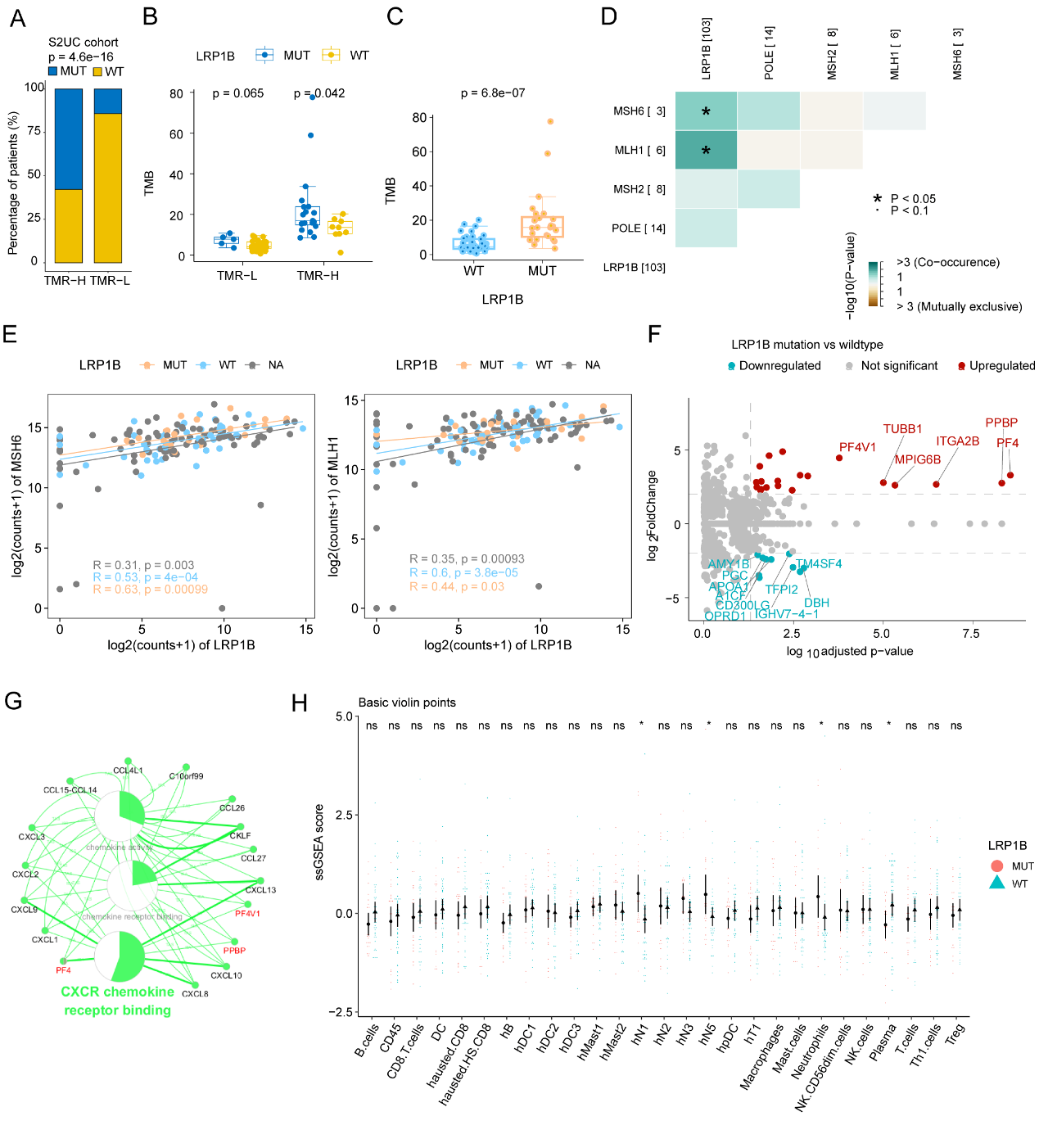


**Figure S5. The association of LRP1B with TMB and immune in S2UC-MARK validation cohort.** (A) Bar plot depicting the percentage of patients with mutant and wild-type *LRP1B* in S2UC-MARK cohort. Two-sided Fisher’s test was used to compare frequencies in the high- and low-TMR score groups. (B) Boxplot showing the TMB in TMR-H and TMR-L groups classified by LRP1B mutation or not. (C) The comparison of TMB between patients with mutant and wild-type *LRP1B.* (D) Heatmap showing the co-occurrence or exclusive effect of LRP1B and DNA mismatch repair genes (*MSH6*, *MSH2*, *MLH1*, *POLE*) (n = 306). * represents *p* < 0.05. (E) Pearson correlation analysis of *LRP1B* and *MSH6/MHL1* gene expression shown as log2(count + 1) (n = 152). Yellow points represent *LRP1B* mutant patients. Blue points mean *LRP1B* wild-type patients. Grey points represent patients without *LRP1B* genotyping. (F) The volcano plot showing the differentially expression genes between patients with mutant *LRP1B* or not (n = 65). (G) The enrichment pathway of differentially expressed genes is visualized by ClueGo/CluePedia plugin of Cytoscape. (H) The comparison of ssGSEA score of gene expression markers of immune cell infiltrate between mutant and wild-type *LRP1B* patients. Mann-whiteney U test was used for statistical analysis with two-sided tail. * means p < 0.05.
